# Efficacy of Short Novel Antimicrobial Peptides in a Mouse Model of *Staphylococcus pseudintermedius* Skin Infection

**DOI:** 10.3390/antibiotics13060508

**Published:** 2024-05-30

**Authors:** Mingyu Ouyang, Fangrong Wu, Changmin Hu

**Affiliations:** College of Veterinary Medicine, Huazhong Agricultural University, Wuhan 430070, China; mingyuouyang@webmail.hzau.edu.cn (M.O.); wufangrong@webmail.hzau.edu.cn (F.W.)

**Keywords:** antimicrobial peptides (AMPs), *Staphylococcus pseudintermedius*, ADD-A, mouse skin infection model

## Abstract

As the clinical application of antibiotics for bacterial skin infections in companion animals becomes increasingly prevalent, the issue of bacterial resistance has become more pronounced. Antimicrobial peptides, as a novel alternative to traditional antibiotics, have garnered widespread attention. In our study, synthetic peptides ADD-A and CBD3-ABU were tested against *Staphylococcus pseudintermedius* skin infections in KM mice. ADD-A was applied topically and through intraperitoneal injection, compared with control groups and treatments including CBD3-ABU, ampicillin sodium, and saline. Wound contraction, bacterial counts and histology were assessed on days 3 and 11 post-infection. ADD-A and ampicillin treatments significantly outperformed saline in wound healing (*p* < 0.0001 and *p* < 0.001, respectively). ADD-A also showed a markedly lower bacterial count than ampicillin (*p* < 0.0001). Histologically, ADD-A-applied wounds had better epidermal continuity and a thicker epidermis than normal, with restored follicles and sebaceous glands. ADD-A’s effectiveness suggests it as a potential alternative to antibiotics for treating skin infections in animals.

## 1. Introduction

As the number of pets increases, canine pyoderma caused by *Staphylococcus pseudintermedius* (*S. pseudintermedius*) is on the rise [1]. *Staphylococcus pseudintermedius* is a coagulase-positive bacterium that belongs to the *Staphylococcus intermedius* group. It shows significant microbiological similarities to *Staphylococcus aureus* in humans. *S. pseudintermedius* is particularly adept at colonising the skin and mucous membranes of dogs, making it an important part of their natural microbial flora [2]. Studies show that *S. pseudintermediu* colonises approximately 90% of healthy dogs. However, infections can also occur when the host’s immune system is compromised or when there is trauma to the skin [3,4,5,6]. Antibiotic resistance in *S. pseudintermedius* in companion animals primarily results from genetic mutations and antibiotic misuse. Genetic changes alter target sites, while improper use encourages the survival and spread of resistant bacteria [7,8,9]. This results in recurrent and persistent infections and even human infections, posing a serious threat to human health [10].

Due to the reliance on specific antimicrobial mechanisms, traditional antibiotic therapy is not always effective against the increasingly complex mechanisms of resistance, and the search for antibiotic alternatives has become urgent.

Antimicrobial peptides (AMPS), as one of the main components of natural immunity, are increasingly being researched and applied because of their broad spectrum, high efficiency, selective toxicity, stability, and low susceptibility to drug resistance. In chronic wounds, commonly infected by *Staphylococcus aureus* and *Pseudomonas aeruginosa* [11], antimicrobial peptides prove effective, overcoming antibiotic resistance issues [12]. For instance, Brilacidin, a synthetic defensin mimetic, has successfully completed Phase 2 clinical trials targeting human infections. The first study (NCT02052388) focused on severe skin infections, encompassing a broad spectrum of bacterial pathogens, including antibiotic-resistant strains. The second study (NCT01211470) specifically addressed acute bacterial skin infections caused by *Staphylococcus aureus*, including methicillin-resistant *Staphylococcus aureus* (MRSA). Both trials aimed to evaluate the efficacy and safety of Brilacidin in treating these serious human skin infections. The strategic combination of AMPs with antibiotics proves to be an effective tactic against drug-resistant pathogens, aiding in the prevention of resistance emergence and amplifying the effectiveness of antibiotics [13,14,15]. This strikes a balance between efficacy and a favourable safety and resistance profile.

ADD-A(RLYLRIGRR-NH2) is designed and synthesised based on the defensin of the beetle *Allomyrina dichotoma*, while CBD3-ABU (KαWNLRGSαREKαIKNEKLYIFαTSGKLαα LKPK) is designed based on canine defensin. In this study, we selected ADD-A and CBD3-ADU peptides for their promising antimicrobial properties. ADD-A, a synthetic 9-mer peptide derived from an insect defensin active site, was designed with increased positively charged residues to enhance its interaction with microbial membranes [16]. CBD3-ADU, derived from canine β-defensin-3 (cBD3), was chosen for its potent activity and stability [17]. The extended C-terminus of cBD3 offers protection against degradation, making it more stable than other subtypes [18]. Despite the minimal impact of disulfide bonds on β-defensin antimicrobial activity, these bonds create synthesis challenges and high costs [19]. Modifications to ADD-A increased its membrane-disruptive capabilities [16,20], while CBD3-ADU was engineered for enhanced stability and potency. These peptides exhibit resistance to proteolytic degradation, supporting their suitability for therapeutic applications. After modification, these peptides exhibit enhanced antimicrobial activity, including against Gram-positive bacteria, Gram-negative bacteria, and fungi [21].

In vivo, studies have shown that ADD-A does not cause the haemolysis of red blood cells or inhibit the growth of macrophages and fibroblasts. Additionally, these peptides can protect mice from lethal infections through methicillin-resistant *Staphylococcus aureus*. Anti-inflammatory activity studies showed that ADD-A had no effect on the viability of Raw264.7 cells and reduced cytokine expression levels [22]. Yukari Koyama et al. found that ADD-A peptides had a favourable protective effect against endotoxic shock in mice [23], which predicts a high degree of safety of the peptides for in vivo application in animals.

The antimicrobial efficacy of ADD-A and CBD3-ADU was assessed at concentrations of 32 µg/mL and 512 µg/mL. These specific concentrations were selected based on their minimum inhibitory concentrations (MICs) that achieved a 90% inhibition of *S*. *pseudintermedius* in in vitro assay [21,24]. Similarly, ampicillin sodium was used at a concentration of 4.8 µg/mL, which also corresponds to the MIC required to achieve a 90% inhibition of the same bacterial strain. Although ampicillin is not typically used as a topical treatment for *Staphylococcus* skin infections, it was chosen as a comparator antibiotic due to its well-characterised antimicrobial activity. However, previous in vitro antimicrobial assays revealed significant resistance of *S. pseudintermedius* to ampicillin [24]. This finding highlights the necessity of evaluating new antimicrobial agents, such as ADD-A and CBD3-ADU, against strains exhibiting antibiotic resistance.

By selecting these concentrations, we aimed to compare the efficacy of each compound at levels where they demonstrate optimal antimicrobial activity, thus offering a more accurate comparison of their therapeutic potential under conditions that reflect their maximal inhibitory effects.

Although the in vitro and in vivo activity of ADD-A have been investigated, its effects as a topical antibacterial agent in animal models have not been reported. In this study, we investigated the antimicrobial properties and wound-healing capabilities of ADD-A within a murine model of *S. pseudintermedius*-induced skin infection. This research aims to explore the potential of ADD-A as an alternative treatment for canine cutaneous bacterial infections, with broader implications for its application in human medicine.

## 2. Results

### 2.1. Skin Infection Model

#### 2.1.1. Establishment of a Skin *S. pseudintermediu* Infection Model

Table 1 shows that the drug was administered consecutively for seven days after trauma handling of the mice in the experimental group.

After continuously applying the bacterial solution to the wounds of mice for 3 d, it was observed that the wounds exhibited a dark red to black crust formation indicative of haemorrhage and necrosis, accompanied by serous exudate.

#### 2.1.2. Trauma Observation

**Figure 1:**
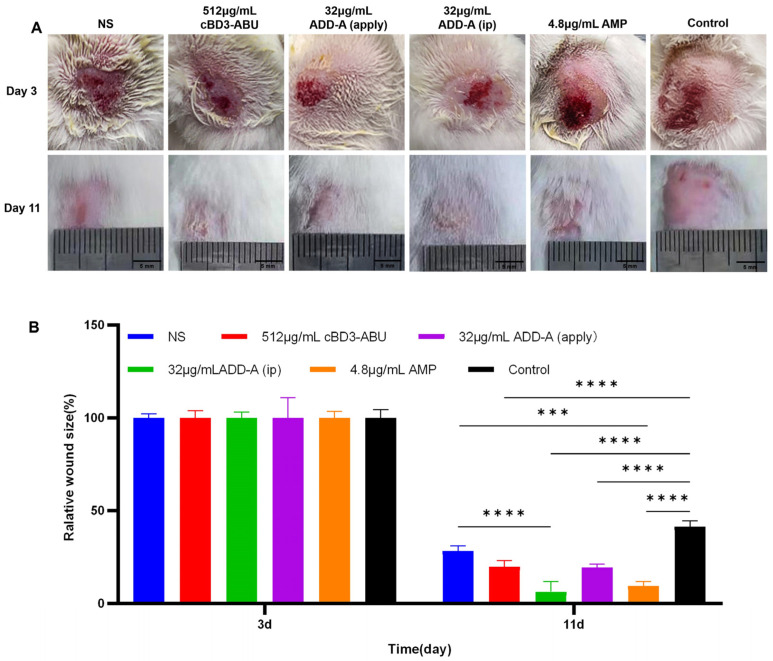
In vivo healing properties of antimicrobial drugs in a skin infection model. (**A**) Representative pictures of wounds infected with *S. pseudintermediu* after treatment with saline, 32 μg/mL ADD-A, 512 μg/mL cBD3-ABU, and 4.8 μg/mL ampicillin sodium on days 3 and 11. (**B**) Wound healing percentage of skin area on days 3 and 11 for each group (n = 3). *** *p* < 0.001 and **** *p* < 0.0001. NS (normal saline), apply (topical application), and ip (intraperitoneal injection). Control refers to untreated samples.

At 11 days, the traumatic area of mice in the saline control group displayed a crimson hue with indented morphology. The traumatic area of the 512 μg/mL cBD3-ABU group exhibited a wound with a central yellowish scab. The traumatic area of the 32 μg/mL ADD-A (apply) group was restored to intact skin, and the hair continued to grow. The traumatic area of the 32 μg/mL ADD-A (ip) group also showed a yellowish scab. The traumatic area of the 4.8 μg/mL ampicillin sodium group had a more intact skin restoration and a vigorous growth of the coat. The trauma control group had a larger hairless area with the presence of a distinctive dark red patchy scab (Figure 1A).

#### 2.1.3. Wound Area Measurement

The statistical results showed that wounds in the treatment groups were generally smaller than those in the control groups, indicating the potential of these treatments to promote wound healing (Figure 1B). At 11 days post-treatment, the wound areas of mice treated with saline (control), 512 μg/mL cBD3-ABU, 32 μg/mL ADD-A (apply), 32 μg/mL ADD-A (ip), and 4.8 μg/mL ampicillin sodium were 28.4%, 19.85%, 6.37%, 19.40%, and 9.50%, respectively. These were all significantly smaller compared to the wound area in the wound control group, which was 41.40% (*p* < 0.01 for the saline control group, *p* < 0.0001 for each antimicrobial treatment group). Among these, 32 μg/mL ADD-A (apply) demonstrated the strongest efficacy in wound healing. Furthermore, the topical application of ADD-A on traumatic wounds was more effective in promoting recovery than its intraperitoneal injection.

### 2.2. Bacterial Enumeration

**Figure 2:**
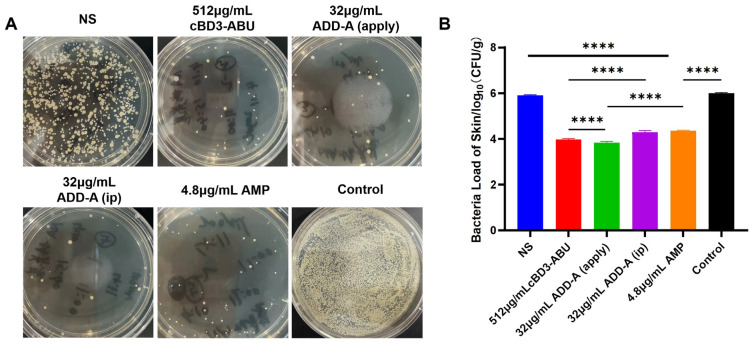
Bacteriostatic effect against *S. pseudintermediu*. (**A**) Representative plate photographs of bacterial colonies after treatment with normal saline (NS) and antimicrobial drugs (ADD-A and CBD3-ADU). The photographs illustrate the comparative effectiveness of each treatment in reducing bacterial colonies. (**B**) Relative bacterial survival of *S. pseudintermediu*. **** *p* < 0.0001. NS (normal saline), apply (topical application), and ip (intraperitoneal injection). Control refers to untreated samples.

The skin bacterial load of infected mice in the trauma control group reached 10^6^ CFU/mL (Figure 2). The antimicrobial drug treatment group showed a 99% killing of *S. pseudintermediu*, while the saline control group did not reduce the bacterial load. On statistical analysis, the number of colonies at the site of infection was significantly lower in the 32 μg/mL ADD-A (apply) group than in the 4.8 μg/mL ampicillin sodium group, with a *p*-value of <0.0001 when comparing these two groups, although an intraperitoneal injection of 32 μg/mL ADD-A also had a good reduction in bacterial load in infected mice (1.31 lg reduction). However, there was a significant reduction in the number of colonies in the 512 μg/mL cBD3-ABU group and the 32 μg/mL ADD-A (apply) group compared to the 32 μg/mL ADD-A (ip) group (*p* < 0.0001).

### 2.3. Hematoxylin-Eosin (HE) Staining Results

**Figure 3:**
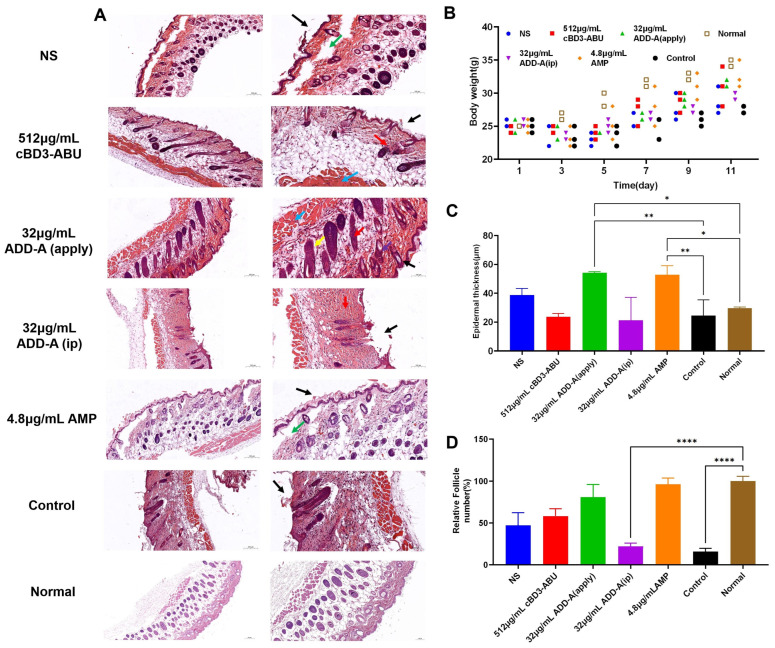
(**A**) Images of H and E-stained skin tissue on day 11 under different treatments. (**B**) Weight changes in mice during recovery. (**C**) The mean epidermal thickness of the wound on day 11. * *p* < 0.05 and ** *p* < 0.01. (**D**) Semi-quantitative statistics of the relative follicle number. The scale bar is 200 μm. **** *p* < 0.0001. NS (normal saline), apply (topical application), and ip (intraperitoneal injection). Control refers to untreated samples.

The histological analysis of wound recovery on day 11 is shown in Figure 3A. In the saline control group and the trauma control group, we observed subepidermal vacuoles in the skin tissues (green arrow). Localised epidermal defects were also visible (black arrow). The epidermis of the skin tissue of the 512 μg/mL cBD3-ABU continuity was better, but the number of sebaceous glands of skin appendages was reduced (red arrow). Complete keratinisation was visible above the epidermis of the skin tissues in the 32 μg/mL ADD-A (apply) group (black arrow), and adipose tissue (yellow arrow), fibrous tissue (purple arrow), and bundles of smooth muscle (blue arrow) could be seen in the dermis in a relatively neat arrangement, and the sebaceous glands of skin appendages (red arrow) were evenly distributed. In the skin tissues of the 32 μg/mL ADD-A (ip) group, local epidermal defects (black arrow) could be seen in the tissue, and the number of sebaceous glands of skin appendages (red arrow) was drastically reduced or even disappeared.

### 2.4. Weight of Mice

The results showed (Figure 3B) that the body weight of mice in all groups decreased after 3 days of dermal bacterial infections but gradually returned to baseline levels during the recovery process. The body weight of mice in the trauma control group increased more slowly than that of the other groups, whereas the body weight of the mice in the 32 μg/mL ADD-A (apply) group and the ampicillin sodium group increased more rapidly, and the overall recovery was better. Despite the variations in recovery speeds, the statistical analysis revealed no significant differences in overall body weight changes among the groups, indicating the high in vivo biosafety of cBD3-ABU and ADD-A.

### 2.5. Epidermal Thickness

The statistics showed (Figure 3C) that the epidermis of the 32 μg/mL ADD-A (apply) group and the ampicillin sodium group was significantly thicker than that of normal mice (*p* < 0.01), and also significantly thicker than that of the trauma-control group (*p* < 0.05). The epidermis of the mice of the trauma-control group and the 32 μg/mL ADD-A (ip) group were of different thicknesses, with large variations in various places. The epidermal thickness in the topical 32 μg/mL ADD-A (apply) group was more consistent across different regions compared to the varied thickness observed in both the trauma-control and 32 μg/mL intraperitoneally injected ADD-A groups.

### 2.6. Relative Follicle Number

By comparing with the number of follicles in the skin of normal mice (Figure 3D), the follicle regeneration rate of the 32 μg/mL ADD-A (apply) group and the ampicillin sodium group reached 96.32% and 80.98%, whereas the number of follicles of the 32 μg/mL ADD-A (ip) group and trauma-control group only remained at 22.09% and 15.95%, which was a drastic reduction in follicles. This demonstrates a significant reduction in follicles compared to the normal group, with *p* < 0.0001 indicating a highly significant difference.

## 3. Discussion

Bacterial skin disease is one of the major diseases affecting the skin of dogs and cats. The main pathogens implicated in bacterial dermatosis in dogs and cats include pyogenic bacteria such as *S. pseudintermediu*—predominantly in dogs—*Staphylococcus epidermidis*, and *Staphylococcus aureus* [25]. These bacteria infect and proliferate on the skin, leading to dermatological conditions. The misuse of antibiotics has made canine bacterial dermatoses increasingly difficult to treat clinically, and the search for new therapeutic options is becoming increasingly urgent.

In this experiment, 32 μg/mL of ADD-A was applied as the therapeutic concentration, which showed a strong effect against *S. pseudintermediu* infection in mice. On the 11th day of infection, the count of *S. pseudintermediu* in the traumatic tissue of the ADD-A group was significantly lower than that of the ampicillin sodium group, and the epidermis was significantly thickened with better continuity; the tissues of each layer were arranged neatly, there was no proliferative fibrous tissue, and the number of follicles and other appendages was almost restored to normal.

Research data showed that the wound healing ability of the antimicrobial peptide was comparable to that of ampicillin sodium, while the antimicrobial effect was stronger than that of ampicillin sodium, suggesting that *S. pseudintermediu* may be resistant to ampicillin sodium. As a natural insect active antimicrobial peptide, ADD-A has a net positively charged amphiphilic structure [26]; due to the negatively charged microbial cell membranes, cationic AMPs can easily accumulate on the cell membranes by electrostatic affinity, which can lead to membrane cleavage and release of cellular contents [27,28]. Based on previous studies, the mechanism of action of ADD-A involves disrupting the bacterial cell membrane, leading to cell lysis or increased membrane permeability. This action allows antimicrobial agents to penetrate the cell more easily, thereby enhancing their bactericidal effect. ADD-A has been shown to exhibit significant antimicrobial activity against *S*. *pseudintermedius* and effectively inhibit biofilm formation by this pathogen [21]. Furthermore, evidence suggests the involvement of alternative antimicrobial mechanisms, which operate by targeting intracellular molecules, namely deoxyribonucleic acid (DNA) and proteins. The disruption of related biological processes and pathways by these mechanisms plays a crucial role in exerting an antimicrobial effect [29]. The sophisticated antimicrobial mechanisms of antimicrobial peptides contribute to the difficulty in developing resistance against them [30,31]. These peptides employ a variety of strategies to combat microbial pathogens, thereby diminishing the potential for the emergence of resistant strains [32].

However, the antimicrobial activity of AMPs is determined by their structural characteristics, including size, residue composition, charge, conformation, helicity, hydrophobicity, and amphiphilicity [33,34,35,36,37]. Thus, identifying suitable carriers, solvents, or modes of administration is crucial for optimising the clinical antimicrobial efficacy of AMPs. Experimental data indicate that the topical application of the antimicrobial peptide ADD-A more effectively promotes wound healing, restores skin structure, and reduces bacterial load compared to intraperitoneal injection. This increased effectiveness may be attributed to ADD-A’s peptide structure, which is susceptible to hydrolysis by proteolytic enzymes in the body, thereby reducing its activity when administered via intraperitoneal injection. The low proteolytic stability of antimicrobial peptides is a known limitation that can affect their clinical efficacy. Although we have not yet assessed the serum stability of ADD-A in this study, we recognise its critical importance for clinical application. Future research, particularly studies involving combination therapies with antibiotics, will focus on evaluating the serum stability of ADD-A to better understand its potential for therapeutic use.

Consequently, the primary focus of subsequent research should be the investigation of the optimised structure of ADD-A and the exploration of appropriate administration methods to augment its efficacy in animal models. Furthermore, studies have demonstrated that the variance in net charge between anionic lipids found in bacteria and those in eukaryotic cells allows for the selective targeting of antimicrobial peptides (AMPs) to microbial pathogens. This selective targeting can be further refined through the rational design of peptide sequences, which aims to enhance microbial selectivity while minimising toxicity towards mammalian cells [38]. Recent studies provide further evidence that antimicrobial peptides act synergistically with conventional antibiotics to help treat canine skin infections, supporting the use of antimicrobial peptides as novel interventions for the treatment of in vivo clinical infections [39].

## 4. Materials and Methods

### 4.1. Bacterial Isolate

*S. pseudintermediu* is a clinical strain isolated from specimens of dogs at the Veterinary Teaching Hospital of Huazhong Agricultural University.

### 4.2. Antimicrobial Peptides

ADD-A (RLYLRIGRR-NH2), was synthesised by Shanghai Derfin Biotechnology Co., Ltd. Shanghai, China, with 97.49% purity. CBD3-ABU (KαWNLRGSαREKαIKNEKLYIFαTSGKLαα LKPK) was synthesised by Dian Bio Co., Ltd. Wuhan, China, by using solid phase synthesis, with 95.61% purity. The antimicrobial peptides were stored in a −20 °C refrigerator and dissolved in sterile water before use.

### 4.3. Animals

35 SPF KM mice. Males, about 40 days old, weighing 20–25 g, were purchased from the Laboratory Animal Centre of Huazhong Agricultural University. Ethics ID Number: HZAUMO-2022-0175.

### 4.4. Main Reagents

Zoletil 50 was purchased from Qingdao Yulong Seaweed Co., Ltd. Qingdao, China; sodium ampicillin sodium was purchased from Nanjing Wobo Biotechnology Co. Nanjing, China; the culture media were purchased from Qingdao Haibo Biotechnology Co. Qingdao, China.

### 4.5. Preparation of Bacterial Suspensions

Activated bacteria were cultured in TSB liquid medium for 12 h at 37 °C with shaking, and after 10-fold multiplicative dilution, the bacterial solution was plated onto TSA solid medium; after 24 h of incubation at 37 °C, the colony count was performed and the concentration of the original bacterial suspension was deduced. Finally, the concentration was adjusted to 1 × 10^9^ CFU/mL with saline.

### 4.6. Observations on the Bacteriostatic Effect of ADD-A on Mice with S. pseudintermediu Infection Model

#### 4.6.1. Mouse Skin Infection Model

After mice were anaesthetised with Zoletil 50 (50 mg/kg), a site on the back of the mouse was selected to remove hair, disinfected with 75% medical alcohol and the skin was scraped with a spatula until subcutaneous bleeding was observed. Logarithmic growth stage *S. pseudintermediu* 0.1 mL (concentration 1 × 10^9^ CFU/mL) was applied evenly to the wound for 3 d.

#### 4.6.2. Animal Grouping and Handling

Thirty-five mice were randomly divided into 7 groups of 5 mice each, and housed in separate cages in a clean room at 25 °C, with free access to food and water, and acclimated for 5 days. On the 11th day, photos were taken at the same location to ensure experimental accuracy, and the area of the trauma was calculated using Image J software 1.8.0.

#### 4.6.3. Bacterial Enumeration

The mice used in our study were SPF (Specific Pathogen Free) grade and housed in a controlled, clean environment. Given these conditions, the influence of other skin pathogens should be minimal and equally distributed across all experimental groups. Therefore, we attributed the colonies observed on TSA plates primarily to *S. pseudintermedius*. On the 11th day after infection, 3 mice from each group were taken and euthanised to minimise suffering, and the skin of the trauma was collected, weighed to prepare tissue homogenate, gradient diluted to 10^−2^, 10^−3^, 10^−4^, etc., and 100 μL of each was coated on the TSA dishes, then incubated at 30 °C for 48 h, and three replicates were set up. The number of colonies on the TSA dishes reached 20 as the minimum limit, and the number of living bacteria on the epidermis was calculated (colony forming units, CFU), and the epidermal bacterial load was expressed as lgCFU (g), where CFU per gram (CFU/g) is calculated as (number of colonies × dilution factor × volume plated (in mL) × 50)/weight of the epidermal sample (in grams).

#### 4.6.4. Observation of Pathological Tissue Sections

On the 11th day after bacterial infection, 1 mouse from each group was taken and sent to Wuhan Google Biological Company for paraffin embedding and paraffin embedding and Hematoxylin and Eosin (H and E) staining, and high-resolution images of the stained sections were collected using a light microscope to assess.

### 4.7. Statistical Analysis

Data were analysed using GraphPad Prism 9. Results of experiments are expressed as mean ± standard deviation (n = 3), *t*-test was used between the two groups and *p* < 0.05 was considered statistically significant, with * *p* < 0.05, ** *p* < 0.01, *** *p* < 0.001 and **** *p* < 0.0001 indicating increasing levels of statistical significance.

## 5. Conclusions

The results of this study are summarised as follows: (1) ADD-A, cBD3-ABU and ampicillin sodium all significantly promoted wound healing; (2) the topical application of ADD-A had a more pronounced in vivo bacterial inhibitory effect than the intraperitoneal injection of ADD-A; (3) the measurement of the skin bacterial load on the 11th day of the test showed that the two antimicrobial peptides had a significant bacterial inhibitory effect compared with the antibiotic; (4) the topical application of ADD-A helps to restore the damaged epidermal structure. ADD-A not only has good antibacterial properties but also promotes the regeneration of a traumatised epidermis and the restoration of epidermal structure, which makes it an effective potential drug for the treatment of bacterial infections in canine skin.

## Figures and Tables

**Table 1:** Experimental groupings and treatments.

Groups	Quantities	Traumatic Injury	Bacterial Infection	Dosages	Mode of Infection	Antimicrobial Treatment	Route of Administration
Group 1	5	Haemorrhage under the skin	*S. pseudintermediu* bacterial solution	1 mL	Cotton swab application	saline	Cotton swab application
Group 2	5	Haemorrhage under the skin	*S. pseudintermediu* bacterial solution	1 mL	Cotton swab application	512 μg/mL cBD3-ABU	Cotton swab application
Group 3	5	Haemorrhage under the skin	*S. pseudintermediu* bacterial solution	1 mL	Cotton swab application	32 μg/mL ADD-A	Cotton swab application
Group 4	5	Haemorrhage under the skin	*S. pseudintermediu* bacterial solution	1 mL	Cotton swab application	32 μg/mL ADD-A	intraperitoneal injection
Group 5	5	Haemorrhage under the skin	*S. pseudintermediu* bacterial solution	1 mL	Cotton swab application	4.8 μg/mL ampicillin sodium	Cotton swab application
Group 6	5	Haemorrhage under the skin	Not handled				
Group 7	5	Not handled					

Note: Group 1 is saline, Group 2, Group 3, Group 4 and Group 5 are antimicrobial drug test groups, Group 6 is an untreated trauma control group and Group 7 is a normal group.

## Data Availability

The data underlying this article are available in the article and in its online Supplementary Materials.

## Supplementary Materials

The following supporting information can be downloaded at: https://www.mdpi.com/article/10.3390/antibiotics13060508/s1, Table S1: Original Data.
